# Food cravings are associated with increased self‐regulation, even in the face of strong instigation habits: A longitudinal study of the transition to plant‐based eating

**DOI:** 10.1111/aphw.12629

**Published:** 2024-12-16

**Authors:** Blair Saunders, Marina Milyavskaya, Kimberly R. More, Jo Anderson

**Affiliations:** ^1^ Division of Psychology University of Dundee Dundee UK; ^2^ Department of Psychology Carleton University Ottawa Canada; ^3^ Department for Health University of Bath Bath UK; ^4^ Faunalytics San Diego CA USA

**Keywords:** behaviour change, complex health behaviour, diet, habit, self‐regulation

## Abstract

Frequently engaging in a positive health behaviour, like following a vegetarian or vegan (veg*n) diet, can bring benefits to both the individual and society. We investigated the roles of two psychological determinants of behaviour—instigation habits and self‐regulation strategy use—in a cohort of individuals who were newly transitioning to a veg*n diet. In a longitudinal study over 6 months (7 waves including baseline), 222 individuals transitioning to a veg*n diet reported their monthly habit strength, craving frequency, self‐regulation strategies and animal product consumption. Our results supported the benefits of having a healthy habit, as stronger habits predicted fewer cravings and lower consumption of animal products, in line with the person's target diet. However, in contrast to some theoretical accounts, having a strong instigation habit did not reduce the use of self‐regulation strategies; people with strong habits used multiple strategies to maintain their diet, especially when they experienced frequent diet‐inconsistent cravings. These findings challenge the idea that habits eliminate the need for self‐regulation, and suggest that habits do not fully circumvent motivational challenges in the pursuit of complex health behaviours. Our results are consistent with recent suggestions that automatic and intentional processes act simultaneously during the enactment of complex health behaviours.

## INTRODUCTION

Vegan and vegetarian (veg*n) diets are increasingly popular and are adopted for a range of ethical, environmental and health reasons (Bryant, 2019; Clem & Barthel, 2021). Veg*n diets are associated with potential protective influences on physical health, including lower rates of non‐communicable diseases, such as heart disease and certain types of cancer (Dinu et al., 2017). Beyond physical health, veg*n diets can positively influence multiple of the World Health Organisation's Quality of Life domains, encompassing psychological (e.g., positive self‐concept through morally consistent actions), social (e.g., social identification with veg*n in‐group members) and environmental factors (e.g., reduced ecological impact, Hargreaves et al., 2021). Yet, adopting and maintaining a veg*n diet is not easy. Negative beliefs about the palatability of a meat‐free diet are associated with a lower probability of adoption (Rosenfeld & Tomiyama, 2020), and plant‐based diets frequently lapse (Faunalytics, 2016). Thus, it is increasingly important to understand the psychological processes that underlie successful dietary transitions. We investigated the independent and combined roles of two psychological determinants of behaviour—instigation habits and self‐regulation strategy use—in a cohort of individuals who were transitioning to a veg*n diet. We tested whether people with strong habits for making diet‐consistent choices experienced fewer diet‐inconsistent cravings, and if developing such a habit means that a person relies on fewer self‐regulatory strategies to resist their cravings.

Instigation habits can promote health and well‐being by automatically prompting individuals to engage in positive behaviours through routinely repeating the behaviour after exposure to a consistent cue (Gardner, 2022; Hagger et al., 2023; Wood & Neal, 2007). When ordering in a restaurant, for example, someone following a plant‐based diet might habitually choose a vegetarian option, resulting in diet‐consistent behaviour. Instigating behaviour habitually is of benefit to the individual because this process is relatively automatic, with the behaviour being the default choice. Thus, habitually instigated behaviours are less susceptible to fluctuations in current motivation, decision‐making capacity or willingness to control the impulse (Wood et al., 2022; Wood & Rünger, 2016). Alternatively, a person without such a habit might take‐in all the menu options, and weigh these against their goals. This goal‐directed process might often result in the same behavioural outcome as the habit‐mediated process. By considering all available options, however, the person also places themself in the way of temptation and might either successfully self‐regulate a craving (e.g., by reminding themselves of the reasons for their veg*n diet), or may fail to do so and succumb to their desire. This latter example highlights the potential fallibility of relying on momentary self‐regulation, and findings from contemporary habit research indicate that people rely less on these goal‐directed processes once habits become established (Monge‐Rojas et al., 2021; Overmeyer et al., 2020; Wood et al., 2022).

While many studies indicate that people use less intentional, goal‐directed processes as habit strength increases (Overmeyer et al., 2020; Triandis, 1977; Zhang et al., 2022), one study recently discovered that this effect might be moderated by the complexity of the targeted behaviour (Saunders & More, 2024). Complex behaviours are commonly studied in health psychology and behavioural medicine (e.g., diet change, exercise uptake, multi‐medication routines), and are more effortful, time‐consuming and multifaceted (i.e., involving more steps) than simple behaviours (Mullan & Novoradovskaya, 2018; Phillips & Mullan, 2022). Saunders and More (2024) observed that people use more self‐regulatory strategies—tactics that can be implemented to increase the likelihood of goal‐consistent behaviour (Duckworth et al., 2018; Hennecke & Bürgler, 2020; Milyavskaya et al., 2021)—for increasingly complex behaviours. More importantly, this wider self‐regulatory repertoire for complex behaviours was true irrespective of habit strength for the same behaviour. Having a wider self‐regulation strategy repertoire (or ‘toolbox’, cf. Fujita et al., 2020) is putatively beneficial because it affords the person a wide range of tools to flexibly draw from in order to up‐ or down‐regulate motivational impulses by changing their environment, attention, cognitive evaluations or behavioural responses (Friese et al., 2024; Hennecke & Bürgler, 2020; Werner & Ford, 2023). Thus, the findings from Saunders and More (2024) suggest that this wider repertoire might support the implementation of complex behaviours in parallel to the development of strong instigation habits.

Nutrition‐related behaviours, such as adopting a veg*n diet, are complex in that they require skill and knowledge of food preparation, and often involve multiple procedural steps which can be effortful and time‐consuming (e.g., meal planning, grocery shopping, food preparation; Phillips et al., 2019). Replicating Saunders and More (2024), we first expected that a wide range of self‐regulation strategies would be implemented in the context of enacting a veg*n diet, even for individuals who have strong instigation habits. In addition, the previous study by Saunders and More did not explore the reasons *why* people with strong instigation habits might nevertheless also use more self‐regulation strategies in the context of complex health behaviours. Here, we tested whether people engage a wider repertoire of self‐regulation strategies in response to the frequency of motivational impulses that they experience in conflict with their dietary goal (i.e., cravings for animal products), even if their veg*n food consumption is habitually instigated. Cravings reflect strong desires in which stimuli with motivational significance to the individual—a stimuli associated with anticipated pleasure or relief—comes to the focus of attention (Hofmann & Van Dillen, 2012; Kavanagh et al., 2005; May et al., 2015). In this sense, cravings are similar to—and likely share dopaminergic neurobiological underpinnings with—the concept of ‘wanting’ in incentive salience theory, in which a previously conditioned cue (e.g., a meat advertisement) triggers reward‐seeking motivation (Olney et al., 2018). Cravings are closely tied to approach motivation for the object of desire but are not necessarily associated with a one‐to‐one mapping with consumption. A person might not act on a craving because the context does not allow for (or strongly disincentivises) consumption, or if the person exerts self‐regulation to stop themselves from acting on their cravings (Kavanagh et al., 2005; Kotabe & Hofmann, 2015; Lopez et al., 2021).

Potential interactions between craving and habit are of particular interest when considering complex health behaviours that people enact across multiple contexts and in dynamic environments. We predicted that people with strong instigation habits will experience less frequent cravings for diet‐inconsistent foods; someone with a strong instigation habit for veg*n foods should select plant‐based products directly and automatically in context, without engaging in a decision‐making process that could stoke temptations. This direct cueing of behaviour is a key benefit of developing habit‐mediated instigation, where context cues directly trigger behaviours regardless of fluctuations in motivation (Wood & Neal, 2007). Our prediction is also consistent with experimental evidence that longer‐term food restriction results in the unlearning of past appetitive cravings and consumption behaviours (Meule, 2020). Thus, as the transition to a veg*n diet is necessarily restrictive, we predicted that the long‐term development of veg*n instigation habits would be associated with reduced cravings for these animal products.

Adopting a veg*n diet over a six‐month interval is unlikely to be temptation‐free, and even those with a very strong instigation habit might encounter cues to conflicting behaviours. Full adherence to a veg*n diet must occur not only in predictable contexts that allow for habit cues, but also in other, more unpredictable environments (e.g., supermarkets, workplaces and dinners with extended family). Here, especially if a person's habit is relatively new, cues might trigger unwanted habits and related behaviours (cf., Gardner, 2015; Gardner et al., 2020; Triandis, 1977). To stop themselves from acting on old habits, we predicted that people transitioning to a veg*n diet will use self‐regulatory strategies if they experience more frequent cravings, even when they simultaneously possess a strong instigation habit.

In the current study, we examined the interplay between habit, craving and self‐regulation strategy use using an existing large, 7‐wave longitudinal dataset that was collected in collaboration with Faunalytics, a non‐profit conducting research to support animal advocates (Kolbuszewska et al., 2022). Two‐hundred and twenty‐two individuals who were transitioning to a veg*n diet took part and completed a baseline survey followed by 6 monthly surveys. Participants reported on their instigation habit strength for choosing veg*n foods, craving frequency, self‐regulation strategies used to counter cravings and consumption of animal products each month.

This design allows for the comparison of goal‐desire conflicts that putatively underlie the instigation of self‐regulation (Becker et al., 2024; Kotabe & Hofmann, 2015), as well as changes in this process as a function of habit development. At intake, all participants wanted to change their diet to become veg*n (i.e., the goal), and we tested the realisation of this goal through the development of a veg*n instigation habit. This instigation was contrasted with opposing cravings to eat animal products (i.e., the conflicting desire) and the reported consumption of animal products as a measure of self‐regulation failure. This oppositional framework was adopted because veg*n diets are exclusionary in that their success depends upon eating no (or significantly less) animal products, and we expected that people in a new dietary transition would likely struggle with overcoming old unwanted habits to eat animal products. Our methodology comprises secondary analyses on an existing dataset to test the following preregistered hypotheses (https://osf.io/2xs3f/):

Hypothesis 1 Habits at a given time point will predict a lower frequency of self‐reported cravings over the following month, and stronger cravings over the past month will be related to weaker current habits.

Hypothesis 2 Monthly craving frequency will predict increased monthly rates of self‐regulation strategy use. We will then test two competing perspectives on the interaction between the frequency of cravings and instigation habit strength in predicting strategy count:

2.1. If developing a strong habit supplants the need for self‐regulatory processes, we would expect an interaction between craving frequency and instigation habit strength in which there is a significant positive relationship between craving and self‐regulation strategy use for people with low habits, but a flat/nonsignificant relationship between the same two variables for people a strong habit.

2.2. However, if habitual and goal‐directed processes are complementary and often co‐occur, we predict that there will be a significant positive relationship between craving levels and strategy use, even for people who have strong habits.

Hypothesis 3 Cravings and strategy use will interact to predict reduced animal product consumption while controlling for habit. People who use self‐regulatory strategies will exhibit more diet‐consistent behaviour despite cravings, compared to cravings that are not countered by these goal‐directed processes. We predict that there is no 3‐way interaction (between cravings, strategy use and habit).

## METHOD

### Participants and procedure

Individuals who were transitioning to a veg*n diet, or have done so in the past two months, were recruited from North American online sources (e.g., vegetarian resource group newsletters) to participate in a 6‐month long study. After providing informed consent for their participation and completing a brief pre‐screen survey, those who were eligible were sent the baseline survey, followed by 6 monthly surveys. All surveys were completed online and contained questions about participants' experiences of becoming veg*n, including measures of behaviours, motivations, barriers, etc. Participants first indicated their desired diet (vegetarian or vegan), and subsequent questionnaires/surveys were adapted to match this choice. Only the constructs relevant to the present study are described below; the full survey is available on OSF (https://osf.io/bhksj/). Two hundred and twenty‐two participants (67.6% women, M_age_ = 31.4) completed at least one survey (M = 4.86 out of a maximum possible seven surveys), resulting in a total of 1,079 completed surveys used for analyses in the present study. Full details about recruitment, planned sample size, attrition and missing data and other participant characteristics can be found on OSF.

### Measures

#### Veg*n instigation habit

The brief 4‐item self‐report automaticity index (SRAI; Gardner et al., 2012) was administered at each time point. Participants responded to each item (e.g., I choose veg*n food without thinking; participants saw either *vegan* or *vegetarian* based on their chosen diet) on a 5‐point scale ranging from 1 = strongly disagree to 5 = strongly agree.

#### Craving

The frequency of cravings over the past month was assessed using 1 item “In the past month, how often have you had cravings for …” The specific object of craving was *meat/fish* for those whose goal was to be vegetarian, and *animal products* for those whose goal was to be vegan. The 6 response options were *never*, *less than once a week*, *1–3 times a week*, *4–6 times per week*, *daily*, *multiple times a day*. This variable was treated as continuous for the purpose of the analyses.

#### Self‐regulation strategies

Participants reported whether they engaged in several different strategies related to their diet over the past month using a checklist (y/n for each strategy). Nine items specifically targeted regulating cravings (e.g., *Avoided places or situations that might tempt you*; *Distracted yourself from a craving; Changed the way you were thinking about a craving for a food you craved*). This strategy selection was broad enough to span all of the stages of the Process Model of Self‐Control (Duckworth et al., 2016), while also providing a brief assessment that was practical within the larger project. Participants also had the option to enter something else under an option of *other, please specify*; this was included for a total of 10 possible strategies. One further item referred to giving in to a craving (*Made an exception and ate something you craved*), so was not considered for the purposes of our analyses. We counted the number of strategies that were endorsed at each time point as the number of strategies (range: 0–10).

#### Meat/animal product consumption

Participants reported on the frequency of eating five different categories of foods (pork or beef; chicken or turkey; eggs; dairy; fish or seafood) over the past month. The five response options were daily; five or six times a week; between two and four times a week; once a week or less; not at all. These options were re‐coded such that larger numbers represented more consumption. Given that consumption behaviours are expected to differ between those with a vegetarian vs. vegan goal, we computed an average consumption score separately for the two groups – for those with a vegan goal we averaged across all five foods; for those with a vegetarian goal we averaged across three foods (not including dairy or eggs).

#### Analyses

We conducted all analyses according to our preregistered plan. As each participant had up to seven surveys, we conducted mixed models with surveys nested within a person using *lmer* in R (Bates et al., 2015). All analyses included random intercepts, as well as the time point as a fixed effect to control for potential differences in effects over time. All predictor variables are at level 1 (the survey level). As we expect an overall effect of the predictors rather than an effect relative to a person's own mean, we did not person‐centre any of the variables. We first tested each model with random slopes for the key independent variables, but removed them if the model did not converge or if there was some other indication that specifying slopes as random is not warranted. Visual inspection of histograms revealed that two of our variables (craving and consumption) were not normally distributed, however, we proceeded with the analyses as planned without transformation because this maximizes the interpretability of the findings, and also because mixed models are robust to violations of normality (Schielzeth et al., 2020). The full code and output of all the models we attempted can be found at https://osf.io/jk269/.

For the first hypothesis, we tested two models, first with habit at the prior time point (t‐1) as a predictor of cravings (model 1a), and then with cravings reported at a given time point predicting habit at the same time point (model 1b). For hypothesis 2, we first examined the distribution of the strategy count variable to ensure it was not zero‐inflated (in which case we would have used a Poisson distribution instead). We then tested a model with current habit and frequency of cravings predicting strategy count (model 2a), and then added the interaction between habit and cravings (model 2b). Finally, for hypothesis 3, we tested a model with the main effects of current habits, frequency of cravings and strategy use (model 3a). We then added the interaction between cravings and strategy use predicting consumption (model 3b). We also ran a model that includes the 3‐way interaction (habit*craving*strategy use; model 3c) and compared it to a model without the 3‐way interaction (model 3d). Using the BIC from models 3c and 3d, we computed the Bayes Factor to determine which model was superior (to rule out a potential 3‐way interaction).

As a robustness check and to account for potential effects of diet goal (vegetarian vs. vegan), we reran all analyses controlling for diet goal; there were no differences in the results (although there was a main effect of goal type on every outcome). We report the preregistered results (without controlling for goal type) in the manuscript, but include all output from the supplementary analyses in online supplements (on OSF).

## RESULTS

Table 1 reports the descriptive statistics for all the variables. Participants reported overall high levels of habitual behaviour, although this increased over the course of the study (from M = 3.64 at baseline to M = 4.32 at the final follow‐up), and participants used on average 3–4 strategies each month to control their cravings. They also reported relatively low cravings and little consumption of animal products. Intraclass correlations (ICCs) show that just over 50% of the variance in cravings, habits and strategy use is at the between‐person level, suggesting that people are fairly consistent in their reports of these variables. Consumption, however, varied more by occasion (only 26% of the variance at the between‐person level).

**TABLE 1:** Descriptive statistics and correlations.

	M	SD	Range	ICC	1	2	3	4
1. Cravings	2.16	.87	1–6	.50	‐‐	−.33 (−.39; −.28)	.02 (−.04; .08)	.12 (.05; .18)
2. Veg*n habit	3.97	.98	1–5	.56	−.40 (−.51; −.30)	‐‐	.06 (.00; .12)	−.22 (−.28; − .16)
3. Strategies	3.80	2.77	0–10	.63	.44 (.32; .55)	.03 −.10; .16)	‐‐	−.10 (−.16; −.04)
4. Consumption	1.62	.76	1–5	.26	.30 (.17; .42)	−.40 (−.51; −.29	−.18 (−.31; −.05)	‐‐

*Note*: between‐subject correlations are below the diagonal, and within‐subject are above. Monthly variables: Craving = animal product craving frequency; Veg*n Habit = habit strength for vegan/vegetarian consumption; Strategies = count of strategies used to counter cravings; Consumption = frequency of diet‐inconsistent animal product consumption.

**FIGURE 1:**
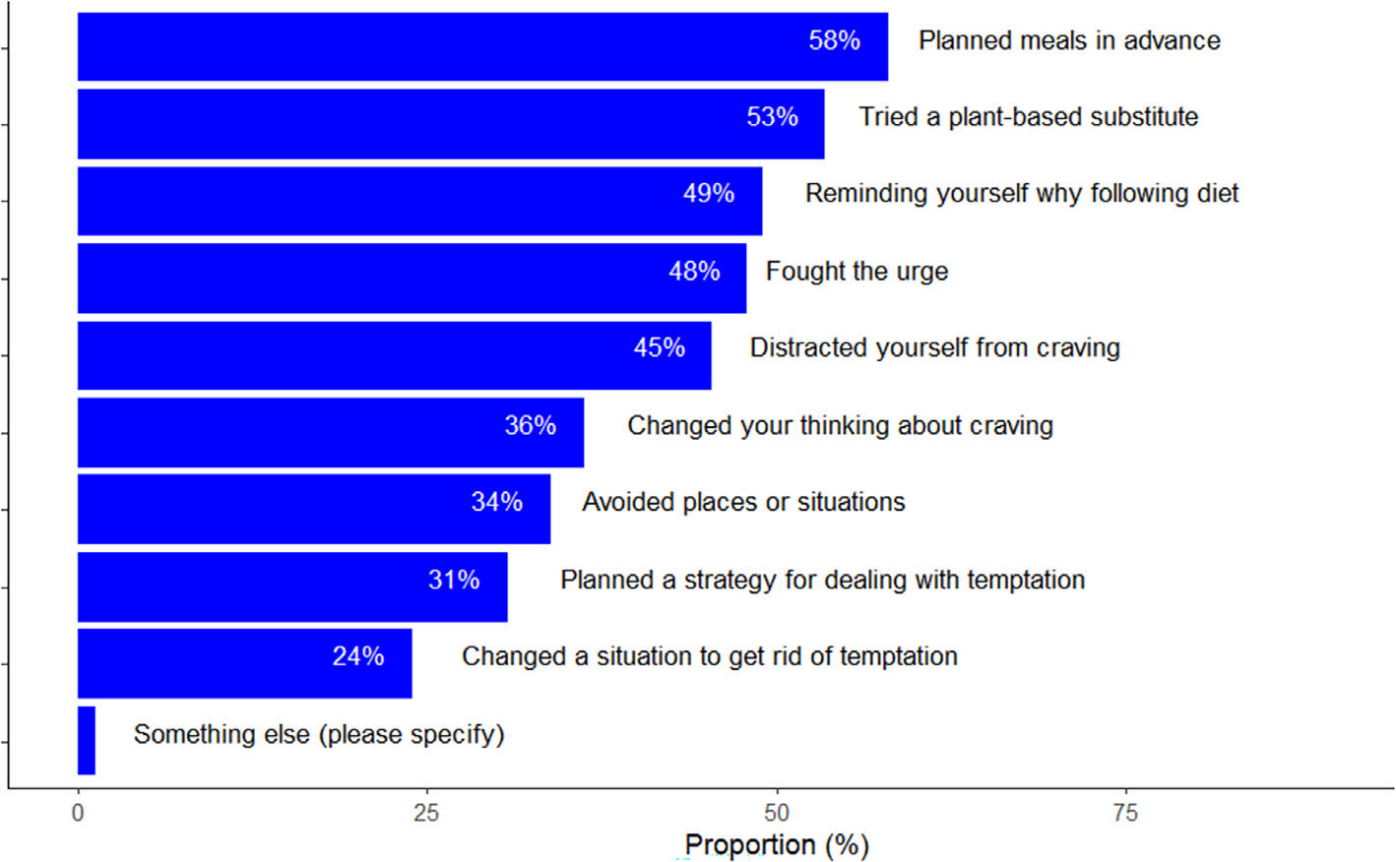
The popularity of strategies used to resist craving.

Among the strategies used by participants, planning meals in advance was the most prevalent (endorsed on 58% of occasions), followed by trying a plant‐based substitute for a craved food. Figure 1 provides proportions for each strategy.

### Confirmatory (preregistered) analyses

We first tested the relation between habit and cravings (Hypothesis 1). Results of model 1a show that stronger current habits led to lower cravings over the subsequent month, *b* = −0.13, 95% CI [−0.19; −0.06], with habits explaining 3% of the variance in cravings. In parallel, the results from model 1b showed that less frequent cravings over the past month were linked to stronger current habits for veg*n consumption (model 1b), *b* = −0.34, 95% CI [−0.40; −0.28]; cravings explained 12% of the variance in habits. These results support our first hypothesis that habits at a given time point will predict a lower frequency of self‐reported cravings over the following month, and stronger cravings over the past month will be related to weaker current habits.

**FIGURE 2:**
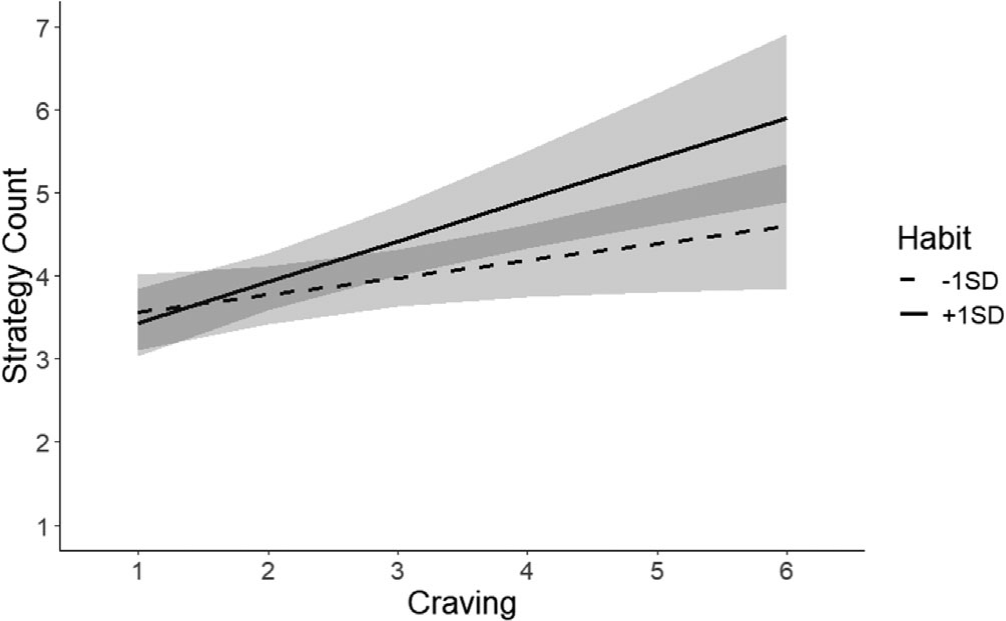
Graph showing the relationship between strategy count and craving frequency for instigation habit strengths one standard deviation above (solid line) and below (dashed line) the mean.

We next tested hypotheses 2.1 and 2.2 concerning the possible interaction between cravings and habits in predicting strategy use. A linear model with fixed effects of habits, cravings, and their interaction predicting strategies found a significant effect of the interaction, *b* = 0.15 [0.01; 0.29], see Table 2.^1^ Figure 2 illustrates this interaction. The relation between cravings and strategies was lower for those with weaker habits. Those with stronger habits used more strategies as they experienced stronger cravings. A follow‐up analysis of only those individuals who reported an instigation habit (an average response of ‘agree’; at least 4 out of 5 on the SRAI scale) found a significant relationship between cravings and strategy use, *b* = 0.42, 95% CI [0.16; 0.69]. This supports our prediction that habitual and goal‐directed processes are complementary processes that co‐occur. However, we did expect that this effect would also be present, and perhaps stronger, for people with weaker habits who would have to rely on self‐regulation strategies to enact their diet. However, as shown in Figure 2, the relationship between craving and strategy use was shallower as habit strength decreased. We found no evidence in any of our models that possessing a strong instigation habit to eat veg*n food was associated with the reduced implementation of self‐regulation strategies at any level of craving, see Figure 2 and Table 2.

**TABLE 2:** Full statistics for models 2a‐3c.

	Model 2a			Model 2b			Model 3a			Model 3b			Model 3c		
*Predictors*	*Estimates*	*Std. error*	*p*	*Estimates*	*Std. error*	*p*	*Estimates*	*Std. error*	*p*	*Estimates*	*Std. error*	*p*	*Estimates*	*Std. error*	*p*
Month	−0.00 (−0.05–0.05)	0.03	0.921	0.00 (−0.05–0.05)	0.03	0.988	**−0.14 (−0.15 – −0.12)**	**0.01**	**<0.001**	**−0.14 (−0.16 – −0.12)**	**0.01**	**<0.001**	**−0.14 (−0.15 – −0.12)**	**0.01**	**<0.001**
Cravings	**0.31 (0.14–0.48)**	**0.09**	**<0.001**	−0.25 (−0.80–0.29)	0.28	0.362	**0.08 (0.03–0.13)**	**0.03**	**0.003**	0.05 (−0.04–0.14)	0.04	0.252	−0.07 (−0.36–0.22)	0.15	0.636
Veg*n habit	0.14 (−0.03–0.31)	0.09	0.111	−0.22 (−0.58–0.15)	0.19	0.241	**−0.11 (−0.16 – −0.06)**	**0.03**	**<0.001**	**−0.11 (−0.16 – −0.06)**	**0.03**	**<0.001**	**−0.18 (−0.35 – −0.02)**	**0.08**	**0.030**
Veg*n habit * cravings				**0.15 (0.01–0.29)**	**0.07**	**0.031**							0.03 (−0.04–0.10)	0.04	0.439
Strategies							−0.01 (−0.02–0.01)	0.01	0.486	−0.02 (−0.07–0.02)	0.02	0.262	−0.18 (−0.37–0.02)	0.10	0.075
Cravings * strategies										0.01 (−0.01–0.03)	0.01	0.358	0.06 (−0.01–0.13)	0.04	0.086
Veg*n habit * strategies													0.04 (−0.01–0.08)	0.02	0.109
(veg*n habit * cravings) *strategies													−0.01 (−0.03–0.00)	0.01	0.139
**Random effects**															
σ^2^	2.30			2.29			0.29			0.29			0.29		
τ_00_	4.19 _id_			4.15 _id_			0.09 _id_			0.09 _id_			0.09 _id_		
ICC	0.65			0.64			0.23			0.24			0.24		
Observations	996			996			996			996			996		
Marginal R^2^/conditional R^2^	0.010/0.650			0.012/0.649			0.219/0.402			0.219/0.403			0.220/0.407		

**Note:** Craving = animal product craving frequency; Veg*n Habit = habit strength for vegan/vegetarian consumption; Strategies = count of strategies used to counter cravings.

Finally, we examined whether habit, cravings, strategies and the interaction between cravings and strategies predicted reported consumption of meat/animal products (hypothesis 3; see Table 2). First, we tested for the main effects of each variable (model 3a); unsurprisingly, habit related to lower consumption, while more cravings were linked with higher consumption. There was no effect of strategies, and, importantly, in the next model testing the interaction between craving and consumption, the interaction was non‐significant (model 3b). This suggests that using more strategies, whether overall or in response to higher cravings, was ineffective in reducing meat consumption. We also tested for the presence of a three‐way interaction between habits, strategies and cravings (model 3c). This interaction was not significant, and follow‐up Bayesian comparison tests showed that the model with no interaction was approximately 467 times better than one with the interaction.

### Exploratory analyses

In addition to studying the overall strategy repertoire, we conducted exploratory analyses to test if our effects hold for both proactive and reactive self‐regulation strategies. The proactive strategies were: *Avoided places of situations that might tempt you*, *Planned a strategy for dealing with temptation* and *Planned Meals in Advance*. The remaining strategies were coded as being reactive, except for *Something else (please specify)* that was left out of these analyses.

We then re‐ran all the models twice, once with the outcome variable of proactive strategy count and then with reactive strategy count (See Tables S1 and S2). Participants reported using an average of 1.23 proactive strategies and 2.56 reactive strategies per month. The use of proactive strategies was not associated with instigation habit strength, *b* = 0.07, 95% CI [−0.01; 0.07] or craving frequency *b* = 0.06, 95% CI [−0.02;0.13]. The interaction between habit and craving remained significant (*b* = 0.07, 95% CI [0.01; 0.13], but simple slope analyses showed that cravings were not associated with more proactive strategies in those who formed a habit (habit score > = 4), *b* = 0.06, 95% CI [−0.02; 0.19]. The same key interaction between instigation habit strength and craving frequency was significant for reactive strategies, *b* = 0.11, 95% CI [0.00; 0.21], with cravings linked to using more reactive strategies in those who had formed a habit, *b* = 0.40, 95% CI [0.23; 0.57].

When examining the effects of proactive and reactive strategies, and their interaction with cravings, on the consumption of animal products (H3), the results are essentially the same as when all strategies are included together (no main effects of strategies or interaction with cravings). However, in the model that included the three‐way interaction (and all two‐way interactions), the three‐way interaction was significant for proactive strategies, *b* = −0.07, 95% CI [−0.11; −0.02]. Follow‐up Bayes factor analyses suggest that this result may not be robust, with the null model (without the interaction) fitting 5x better than the full model with the three‐way interaction. Given these mixed results, and that these were exploratory (non‐pre‐registered analyses), we hesitate to overinterpret this interaction. Complete analyses and results, including a graph breaking down the three‐way interaction, can be found on OSF.

## DISCUSSION

Vegan and vegetarian diets are becoming increasingly popular as ways to address health, sustainability and animal welfare (Bryant, 2019). We investigated the relationships among instigation habits, craving and self‐regulation strategy use over six months during the uptake of a veg*n diet. Having an instigation habit to choose veg*n options predicted reduced cravings in the following month, and was also associated with less diet‐breaking behaviour. These findings are consistent with the putative benefits of forming a healthy habit (Gardner, 2015; Wood & Rünger, 2016). However, we also found that people with strong instigation habits used many self‐regulation strategies, habit strength was not associated with reduced strategy use and the implementation of goal‐directed strategies increased with the frequency of diet‐inconsistent cravings. These findings suggest that having a strong habit to initiate a complex behaviour, such as veg*n eating, does not mean that people rely less on goal‐directed processes to tackle motivational challenges, such as temptation. In fact, the positive relationship between craving frequency and strategy use was stronger for those with stronger instigation habits.

Possessing a habit for a positive health behaviour is assumed to be a boon for the individual (Gardner, 2015). Indeed, our findings that instigation habits both protected against cravings the following month, and predicted less diet‐breaking, are broadly consistent with the theoretically beneficial effects of habits on positive health behaviours, and the specific suggestion that habits are protective against short‐term ‘whims’, such as transient cravings (Wood et al., 2022). However, our results are also consistent with recent suggestions that strong instigation habits for complex behaviours coincide with the use of a wide array of self‐regulation strategies (Saunders & More, 2024). Extending these previous findings, our results identified goal‐conflicting temptations (in this case, food cravings) as a process variable that might explain *why* people with strong instigation habits might nevertheless also implement self‐regulation strategies. Exploratory analyses further suggested that these effects were limited to down‐regulatory, reactive self‐regulation strategies that act on cravings in the moment, rather than proactive strategies that might stop cravings from developing in the first place.

Goal‐directed processes may operate on either an implicit or explicit level (Custers et al., 2019), and the implementation of self‐regulation more specifically could be either deliberate and effortful, or more automatic and effortless (Gillebaart & De Ridder, 2015). Thus, one should consider if implementing self‐regulation in the context of habitual behaviour involves a complete mode‐switch from implicit habitual processes to explicit self‐regulatory processes, or, instead, if people develop automaticity in behavioural habits and self‐regulation simultaneously. Rather than one or the other, however, we suspect that our observed pattern of results could easily reflect a mixture of effortful and effortless self‐regulation. Given that strategies are likely to be most beneficial in unpredictable environments, and people use multiple different strategies to counteract cravings, it seems unlikely that people would have sufficient repeated exposure to stable cues to develop strong habits for all their self‐regulation strategies. However, some self‐regulation might become more automatic if individuals developed if‐then rules, similar to implementation intentions (e.g., “I distract myself whenever I smell meat cooking”; Gollwitzer & Sheeran, 2006; Hagger & Luszczynska, 2014). It is important to note that such ‘habits of thought’ would nevertheless still be considered goal‐directed because they do not involve a cue directly triggering the ideomotor processes that instigate behaviour. One challenge for ongoing research will be developing methods that can validly and reliably differentiate between automatic and intentional self‐regulation during the implementation of complex health behaviours.

The positive relationship between craving frequency and self‐regulation strategy use was unexpectedly stronger for individuals who instigated their veg*n diets more automatically compared to those with weaker instigation habits. This is contrary to our initial thinking that self‐regulation use would be used more to compensate for weak habits. We suspect two possible, not mutually exclusive, explanations. One possibility might be that a willingness or ability to develop strong instigation habits also promotes the development of habitual self‐regulation, as mentioned above. This explanation could be consistent with theories of craving in which reward cues trigger intrusive and elaborated cognitions related to the object of desire (Kavanagh et al., 2005); while such intrusions could represent unwanted appetitive cognitions (i.e., temptations), they may also come to include thoughts intended to counteract the desire (i.e., self‐regulation). A second explanation is that people with weaker habit development were also less committed to their new diet, meaning that they were less likely to counter their cravings with self‐regulation strategies. This interpretation is consistent with other suggestions that habits are quite closely related to the extent with which people identify with, and are, therefore, committed to, the behaviour that is enacted due to their given habit (Rhodes, 2021; Verplanken & Sui, 2019).

While instigation habits for consuming veg*n food predicted increased dietary adherence and reduced cravings, self‐regulation strategies were not associated with successful reductions in unwanted consumption. This finding is in contrast to the theoretical purpose of self‐regulation as a way to control impulses (Carver & Scheier, 1998; Duckworth et al., 2018; Inzlicht et al., 2021), and with recent experience sampling studies in which implementing more self‐regulation strategies was associated with regulatory success (Lopez et al., 2021; Milyavskaya et al., 2021). However, such ESM studies measure self‐regulation implementation on a more immediate timescale and still only report moderate success in stopping a person from acting on a desire. If self‐regulation is deployed mostly in situations where people experience challenges to their diet (e.g., cravings for meat), and is also only moderately successful, these factors could explain the lack of correlation between strategy use and longer‐term dietary behaviour. In contrast, instigation habits develop in response to stable environmental cues that trigger the enactment of the behaviour, perhaps explaining why habits do a better job of predicting dietary success than self‐regulation.

While our results were complementary to previous findings (Saunders & More, 2024), they do not speak to the relationship between these variables and long‐term diet change. Someone who has maintained a vegan diet for several years, for example, might experience significantly fewer cravings for foods containing animal products than someone who has been vegan for less than 6 months. Relatedly, memory traces for old habits (e.g., eating meat) might not have fully decayed in a novice compared to an expert vegan. Consequently, studies investigating the interaction between self‐regulation repertoire and habit would benefit from studying particularly well‐ingrained behaviours either cross‐sectionally or in longer‐duration observational studies. In these studies, it would be beneficial to investigate the trajectory of habits for choosing veg*n and animal‐based foods over time to assess if the emergence of one habit (e.g., to choose plant‐based foods) is associated with the undoing of the opposing habit and the reduced frequency of downregulatory self‐regulation strategies. Similarly, on‐going research could test if those with well‐established veg*n instigation habits use self‐regulation strategies to encourage veg*n choices, rather than focusing on the avoidance of animal products. A finding that habits for complex behaviours commonly coincide with the deployment of a wide range of self‐regulation strategies in those with expertise would provide particularly strong evidence for the common cooccurrence of habit‐mediated and goal‐directed processing.

Our results could be generative in the development of future health behaviour change interventions. As health psychologists are often interested in changing higher‐order, complex health behaviours (e.g., Phillips et al., 2019), our results suggest that on‐going theory and intervention development would benefit from closely considering the potential collaboration between habitual and goal‐directed processes in the instigation and maintenance of everyday positive health behaviours (see also Gardner et al., 2024). Novel interventions could be developed to help individuals to identify times and places where they might experience cravings, as our results suggest that habitual and goal‐directed processes will interact most in those circumstances. Such interventions may not only be significant for the general population seeking to modify their diet but also for patients who have been advised to make dietary changes as part of their medical treatment, such as kidney disease or coronary heart disease (NHS, 2018; NIDDK, 2016). Understanding how to combat cravings for previously enjoyed foods in environments varying in controllability is of utmost importance, as most patients do not adhere to their prescribed diets. For instance, one study found that only 4% of patients report high adherence to their prescribed diabetic diet (Selvam et al., 2023).

One limitation of our study is that we measured self‐regulation repertoire per month rather than measuring the frequency of strategy use. Thus, if one person used a reappraisal strategy every day and another person used this strategy only once per month, then they would have the same score in our count measure. Repertoire is considered an important aspect of self‐regulatory ability that allows a person to adapt flexibly to challenges if they have a large range of tools to draw from (Friese et al., 2024), and, indeed, repertoire predicts successful regulation in other studies (Bürgler et al., 2021; Milyavskaya et al., 2021; Werner et al., 2023). That said, it is also possible that a high self‐regulation strategy repertoire could, in some cases, reflect trial and error learning in which a person is trying and failing to use many strategies. However, it should be noted that strategy count was relatively stable over time within people (intra‐class correlation of .63), suggesting that individuals were not varying strategies intensely from month to month. Nevertheless, ongoing research could use more intensive longitudinal methods (e.g., daily diary or ecological momentary assessment) to more completely understand both how strategy count relates to strategy frequency, and how this relates to momentary levels of self‐regulatory success in the context of instigation habits.

## CONCLUSION

Tracking a cohort of new veg*ns longitudinally across 7‐waves of data collection, we observed that individuals who possess strong instigation habits to consume veg*n foods used multiple self‐regulation strategies to counteract cravings that conflicted with their veg*n dietary goals. These results support an emerging idea that rather than being separable systems that seldomly interact, goal‐directed self‐regulatory processes and habit‐mediated processes might coincide during the enactment of positive health behaviours, such as adopting a new diet. Our results specifically suggest that these strategies might be deployed because a person is frequently experiencing goal‐incongruent cravings.

## Supporting information


**Table S1.** Results of exploratory analysis using only reactive strategies.

**Table S2.** Results of exploratory analysis using only proactive strategies.

## Data Availability

The data that support the findings of this study are openly available Open Science Framework at https://osf.io/jk269/

## ENDNOTE

^1^
We first tested a model (see model 2a) with no interaction, and found that cravings predicted increased strategy use, while habit‐level was not associated with strategy use.
