# Supplementary material for: Food cravings are associated with increased self‐regulation, even in the face of strong instigation habits: A longitudinal study of the transition to plant‐based eating
Source: Appl Psychol Health Well Being. 2024 Dec 16;17(1):e12629. doi: 10.1111/aphw.12629 (PMC11649395; doi:10.1111/aphw.12629)
Supplement: Supplementary file 1 — Table S1. Results of exploratory analysis using only reactive strategies. [file APHW-17-0-s002.docx]

|  | **Model 2a** | | | **Model 2b** | | | **Model 3a** | | | **Model 3b** | | | **Model 3c** | | | |
| --- | --- | --- | --- | --- | --- | --- | --- | --- | --- | --- | --- | --- | --- | --- | --- | --- |
| *Predictors* | *Estimates* | *std. Error* | *p* | *Estimates* | *std. Error* | *p* | *Estimates* | *std. Error* | *p* | | *Estimates* | *std. Error* | *p* | *Estimates* | *std. Error* | *p* |
| survey | -0.01 (-0.05 – 0.03) | 0.02 | 0.792 | -0.00 (-0.04 – 0.04) | 0.02 | 0.875 | **-0.14 (-0.15 – -0.12)** | **0.01** | **<0.001** | | **-0.14 (-0.15 – -0.12)** | **0.01** | **<0.001** | **-0.14 (-0.16 – -0.12)** | **0.01** | **<0.001** |
| cravings | **0.32 (0.19 – 0.45)** | **0.07** | **<0.001** | -0.08 (-0.49 – 0.33) | 0.21 | 0.686 | **0.08 (0.03 – 0.14)** | **0.03** | **0.002** | | 0.06 (-0.02 – 0.14) | 0.04 | 0.152 | 0.07 (-0.21 – 0.35) | 0.14 | 0.620 |
| habit | 0.08 (-0.05 – 0.20) | 0.06 | 0.226 | -0.17 (-0.45 – 0.10) | 0.14 | 0.216 | **-0.11 (-0.15 – -0.06)** | **0.03** | **<0.001** | | **-0.10 (-0.15 – -0.06)** | **0.03** | **<0.001** | -0.12 (-0.27 – 0.04) | 0.08 | 0.141 |
| cravings x habit |  |  |  | **0.11 (0.00 – 0.21)** | **0.05** | **0.043** |  |  |  | |  |  |  |  |  |  |
| reactive strat |  |  |  |  |  |  | -0.01 (-0.04 – 0.01) | 0.01 | 0.313 | | -0.03 (-0.09 – 0.02) | 0.03 | 0.271 | -0.14 (-0.39 – 0.12) | 0.13 | 0.295 |
| cravings x reactive strat |  |  |  |  |  |  |  |  |  | | 0.01 (-0.01 – 0.03) | 0.01 | 0.452 | 0.03 (-0.06 – 0.12) | 0.05 | 0.490 |
| habit x cravings |  |  |  |  |  |  |  |  |  | |  |  |  | -0.01 (-0.08 – 0.07) | 0.04 | 0.882 |
| habit x reactive strat |  |  |  |  |  |  |  |  |  | |  |  |  | 0.02 (-0.04 – 0.08) | 0.03 | 0.432 |
| (habit x cravings) x reactive strat |  |  |  |  |  |  |  |  |  | |  |  |  | -0.00 (-0.03 – 0.02) | 0.01 | 0.675 |
| **Random Effects** | | | | | | | | | | | | | | | | |
| σ^2^ | 1.34 | | | 1.34 | | | 0.29 | | | 0.29 | | | 0.29 | | | |
| τ_00_ | 1.98 _id_ | | | 1.97 _id_ | | | 0.09 _id_ | | | 0.09 _id_ | | | 0.09 _id_ | | | |
| ICC | 0.60 | | | 0.60 | | | 0.23 | | | 0.24 | | | 0.24 | | | |
| N | 208 _id_ | | | 208 _id_ | | | 208 _id_ | | | 208 _id_ | | | 208 _id_ | | | |
| Observations | 996 | | | 996 | | | 996 | | | 996 | | | 996 | | | |
| Marginal R^2^ / Conditional R^2^ | 0.020 / 0.604 | | | 0.022 / 0.604 | | | 0.220 / 0.401 | | | 0.220 / 0.403 | | | 0.220 / 0.405 | | | |

Table S1. Results of exploratory analysis using only reactive strategies.
