# Supplementary material for: Food cravings are associated with increased self‐regulation, even in the face of strong instigation habits: A longitudinal study of the transition to plant‐based eating
Source: Appl Psychol Health Well Being. 2024 Dec 16;17(1):e12629. doi: 10.1111/aphw.12629 (PMC11649395; doi:10.1111/aphw.12629)
Supplement: Supplementary file 2 — Table S2. Results of exploratory analysis using only proactive strategies. [file APHW-17-0-s001.docx]

|  | **Model 2a** | | | **Model 2b** | | | **Model 3a** | | | **Model 3b** | | | **Model 3c** | | |
| --- | --- | --- | --- | --- | --- | --- | --- | --- | --- | --- | --- | --- | --- | --- | --- |
| *Predictors* | *Estimates* | *std. Error* | *p* | *Estimates* | *std. Error* | *p* | *Estimates* | *std. Error* | *p* | *Estimates* | *std. Error* | *p* | *Estimates* | *std. Error* | *p* |
| survey | 0.01 (-0.01 – 0.03) | 0.01 | 0.394 | 0.01 (-0.01 – 0.04) | 0.01 | 0.331 | **-0.14 (-0.15 – -0.12)** | **0.01** | **<0.001** | **-0.14 (-0.16 – -0.12)** | **0.01** | **<0.001** | **-0.14 (-0.15 – -0.12)** | **0.01** | **<0.001** |
| cravings | 0.06 (-0.02 – 0.13) | 0.04 | 0.138 | -0.20 (-0.44 – 0.04) | 0.12 | 0.104 | **0.08 (0.02 – 0.13)** | **0.03** | **0.004** | 0.05 (-0.02 – 0.13) | 0.04 | 0.150 | -0.12 (-0.34 – 0.10) | 0.11 | 0.291 |
| habit | 0.07 (-0.01 – 0.14) | 0.04 | 0.079 | -0.09 (-0.25 – 0.07) | 0.08 | 0.247 | **-0.11 (-0.16 – -0.06)** | **0.03** | **<0.001** | **-0.11 (-0.16 – -0.06)** | **0.03** | **<0.001** | **-0.22 (-0.37 – -0.07)** | **0.08** | **0.003** |
| Cravings x habit |  |  |  | **0.07 (0.01 – 0.13)** | **0.03** | **0.027** |  |  |  |  |  |  |  |  |  |
| proactive strat |  |  |  |  |  |  | 0.00 (-0.04 – 0.04) | 0.02 | 0.996 | -0.04 (-0.15 – 0.07) | 0.06 | 0.441 | **-0.72 (-1.22 – -0.23)** | **0.25** | **0.004** |
| cravings x proactive strat |  |  |  |  |  |  |  |  |  | 0.02 (-0.03 – 0.06) | 0.02 | 0.405 | **0.28 (0.11 – 0.45)** | **0.09** | **0.001** |
| habit x cravings |  |  |  |  |  |  |  |  |  |  |  |  | 0.05 (-0.01 – 0.10) | 0.03 | 0.116 |
| habit x proactive strat |  |  |  |  |  |  |  |  |  |  |  |  | **0.17 (0.05 – 0.29)** | **0.06** | **0.004** |
| (habit x cravings) x proactive strat |  |  |  |  |  |  |  |  |  |  |  |  | **-0.07 (-0.11 – -0.02)** | **0.02** | **0.002** |
| **Random Effects** | | | | | | | | | | | | | | | |
| σ^2^ | 0.49 | | | 0.49 | | | 0.29 | | | 0.29 | | | 0.29 | | |
| τ_00_ | 0.49 _id_ | | | 0.48 _id_ | | | 0.09 _id_ | | | 0.09 _id_ | | | 0.09 _id_ | | |
| ICC | 0.50 | | | 0.49 | | | 0.24 | | | 0.24 | | | 0.24 | | |
| N | 208 _id_ | | | 208 _id_ | | | 208 _id_ | | | 208 _id_ | | | 208 _id_ | | |
| Observations | 996 | | | 996 | | | 996 | | | 996 | | | 996 | | |
| Marginal R^2^ / Conditional R^2^ | 0.005 / 0.504 | | | 0.009 / 0.499 | | | 0.217 / 0.407 | | | 0.218 / 0.405 | | | 0.225 / 0.409 | | |

Table S2. Results of exploratory analysis using only proactive strategies.
